# Supplementary figures and images for: Adaptive representations of sound for automatic insect recognition
Source: PLoS Comput Biol. 2023 Oct 4;19(10):e1011541. doi: 10.1371/journal.pcbi.1011541 (PMC10578591; doi:10.1371/journal.pcbi.1011541)

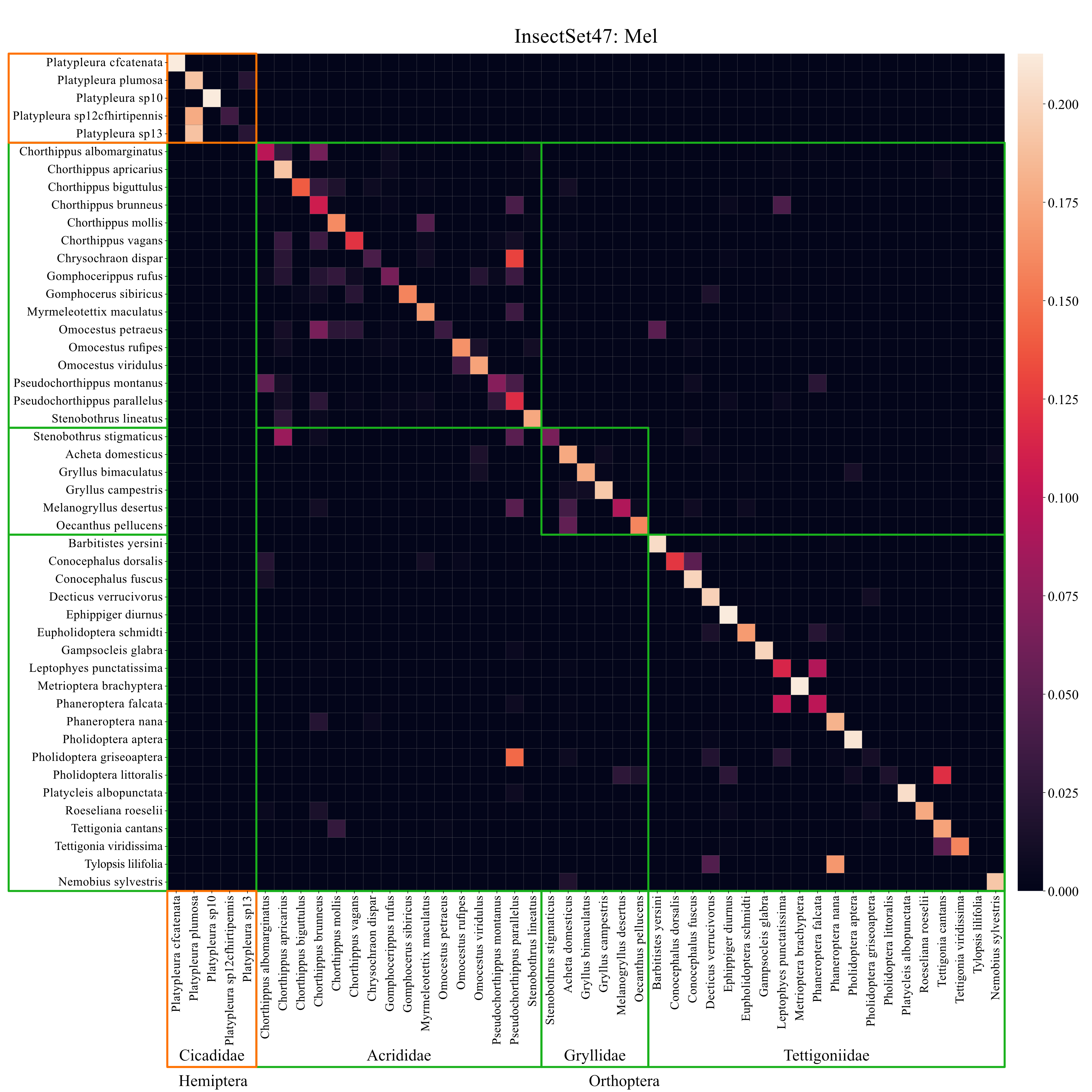

Supplement: S1 Fig — The vertical axis displays the true labels of the files, the horizontal axis shows the predicted labels, grouped into order, family and genus. (TIFF) [file pcbi.1011541.s001.tiff]

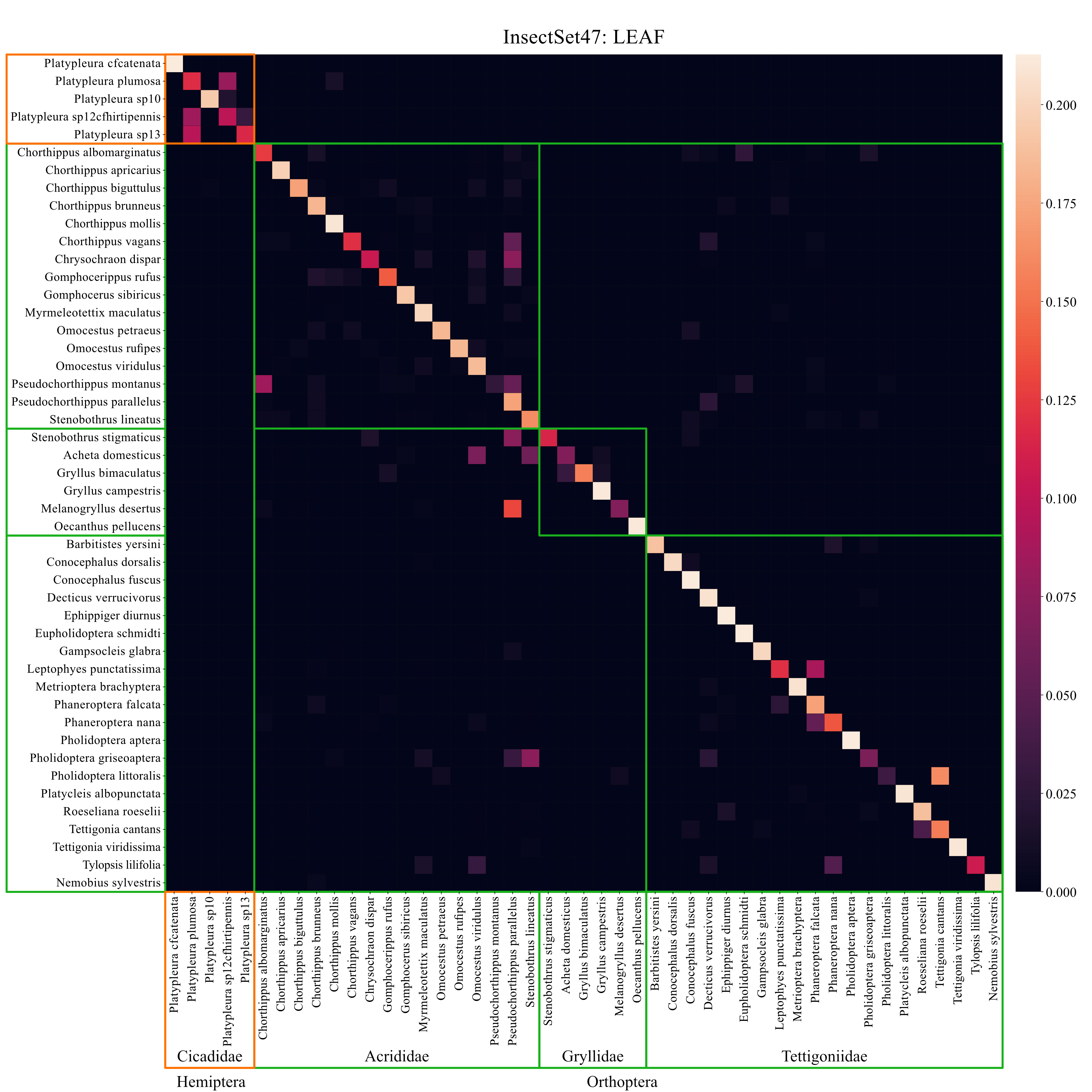

Supplement: S2 Fig — The vertical axis displays the true labels of the files, the horizontal axis shows the predicted labels, grouped into order, family and genus. (TIFF) [file pcbi.1011541.s002.tiff]

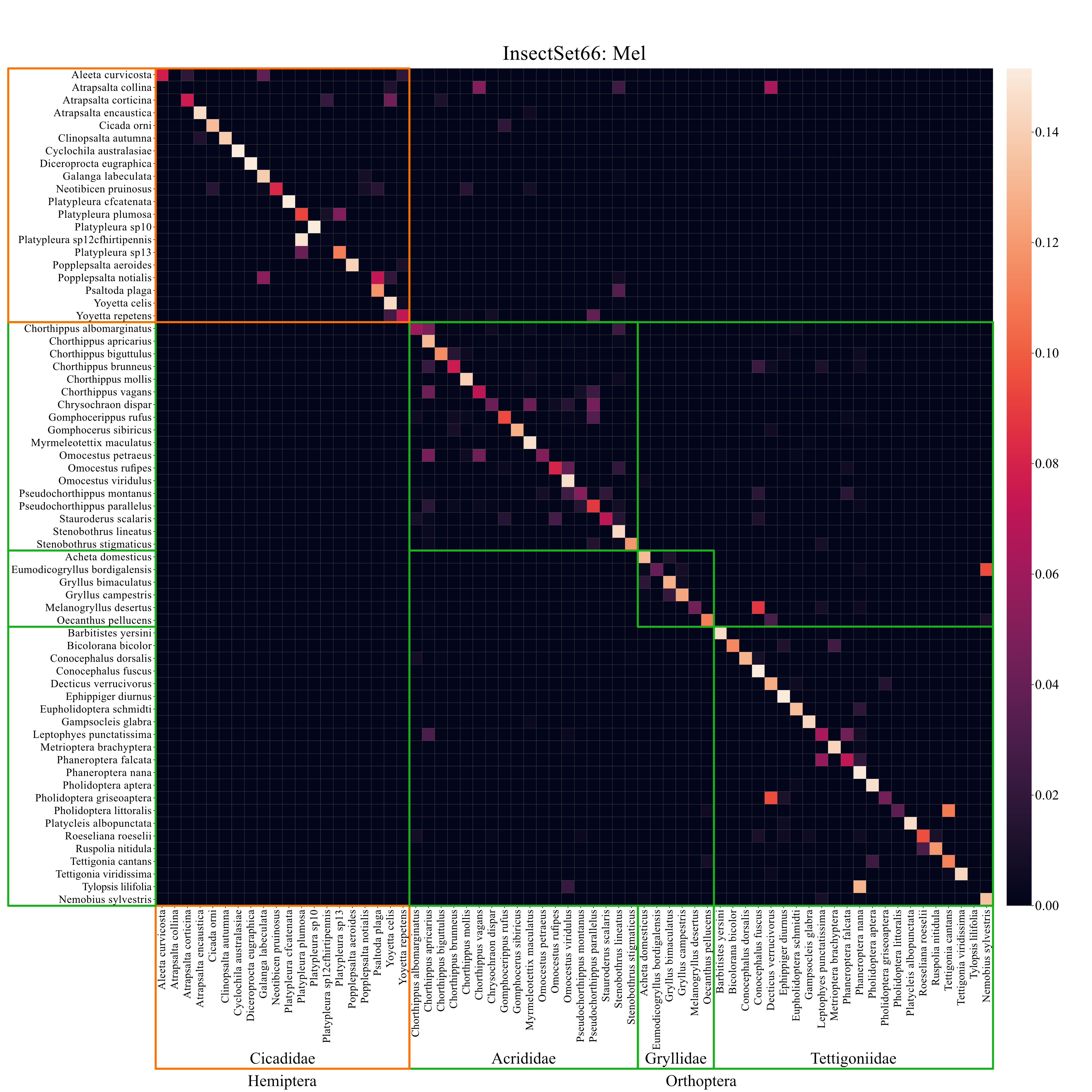

Supplement: S3 Fig — The vertical axis displays the true labels of the files, the horizontal axis shows the predicted labels, grouped into order, family and genus. (TIFF) [file pcbi.1011541.s003.tiff]

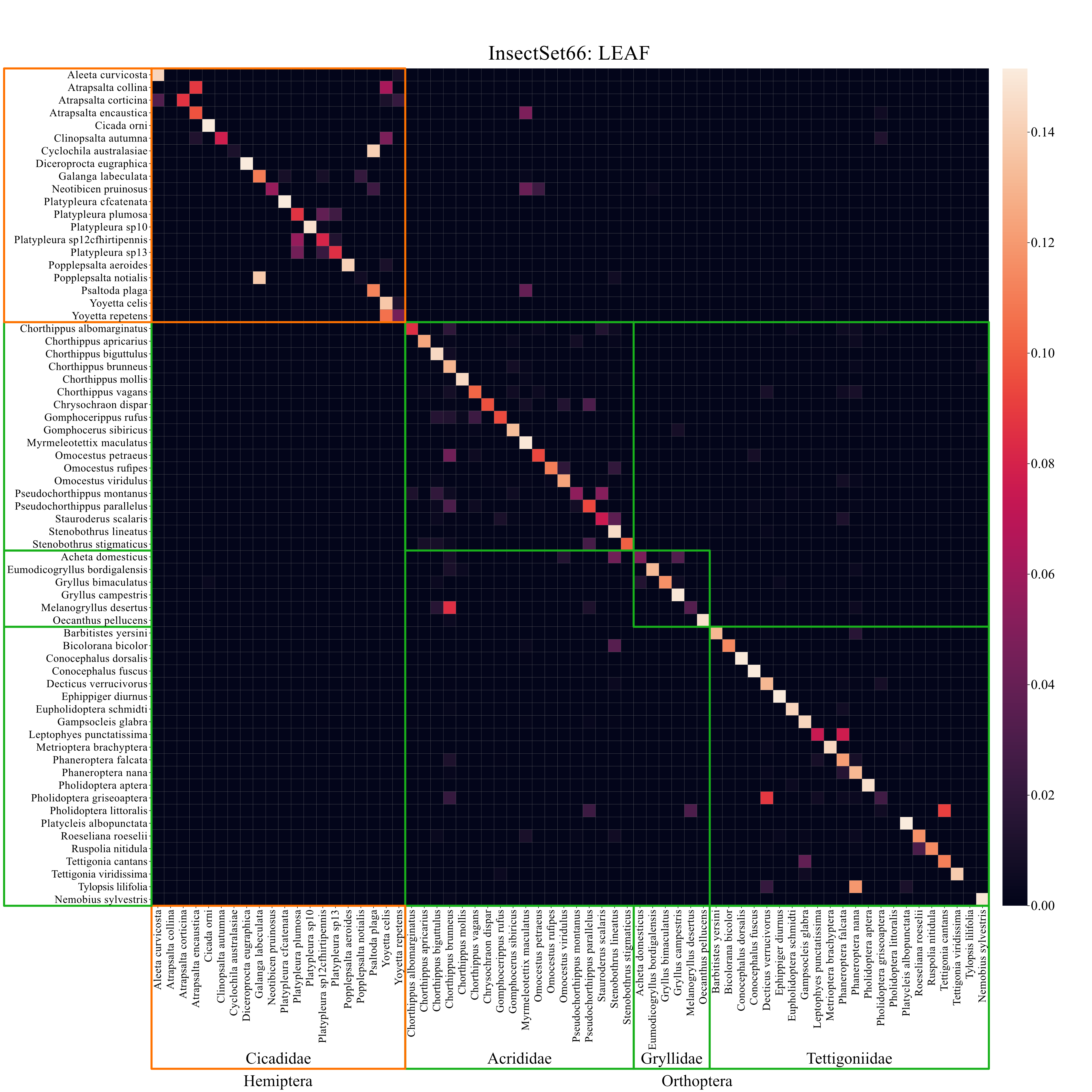

Supplement: S4 Fig — The vertical axis displays the true labels of the files, the horizontal axis shows the predicted labels, grouped into order, family and genus. (TIFF) [file pcbi.1011541.s004.tiff]

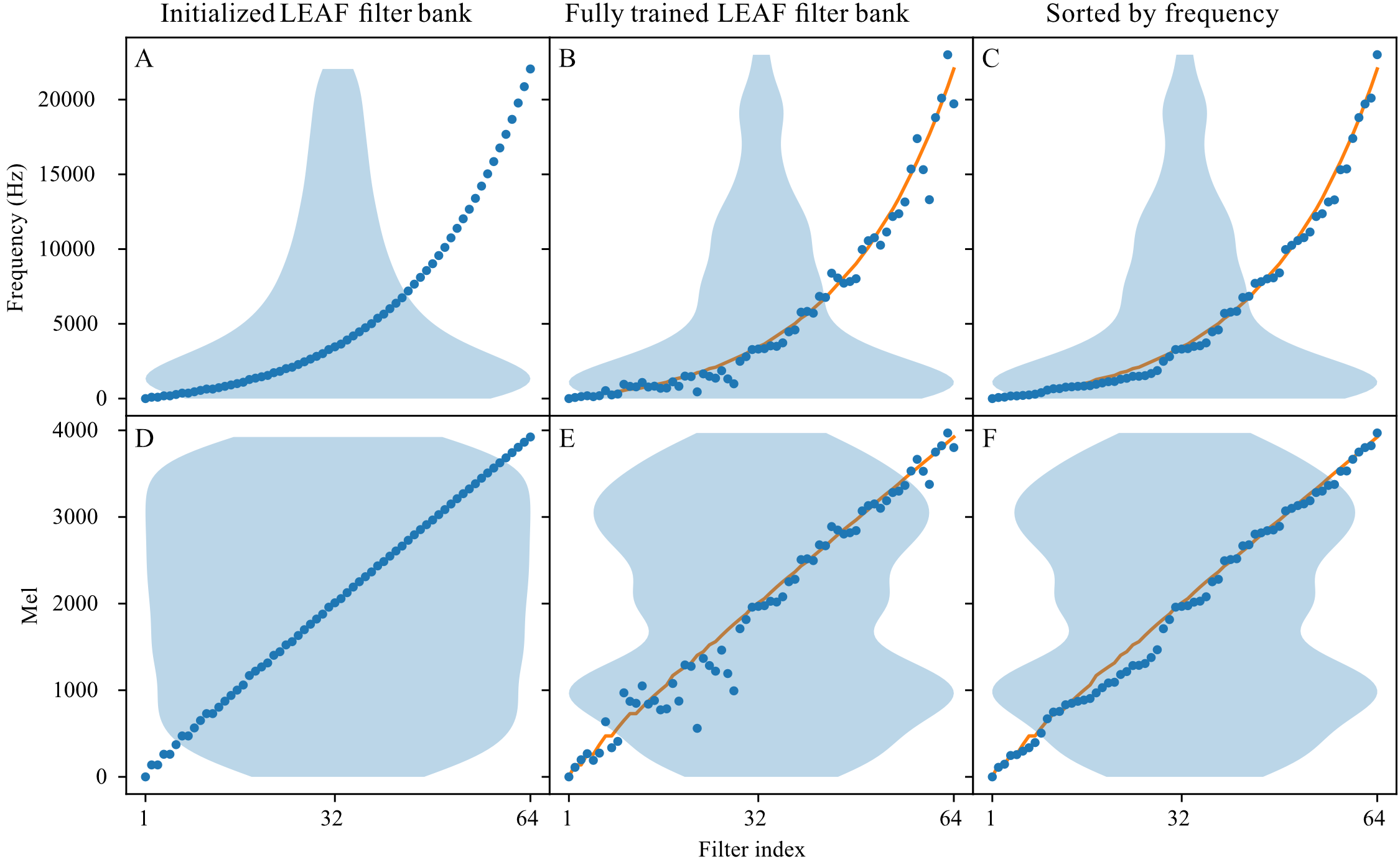

Supplement: S5 Fig — Plots A and D show the initialization curve before training, which is based on the mel scale. Plots B and E show the deviation of each filter from their initialized position after training. Plots C and F show the filters sorted by center frequency, and demonstrate the overall coverage of the frequency range, but do not represent the real ordering in the LEAF representations. Violin plots show the density of filters over the frequency spectrum, the orange line shows the initialization curve for comparison. (TIFF) [file pcbi.1011541.s005.tiff]

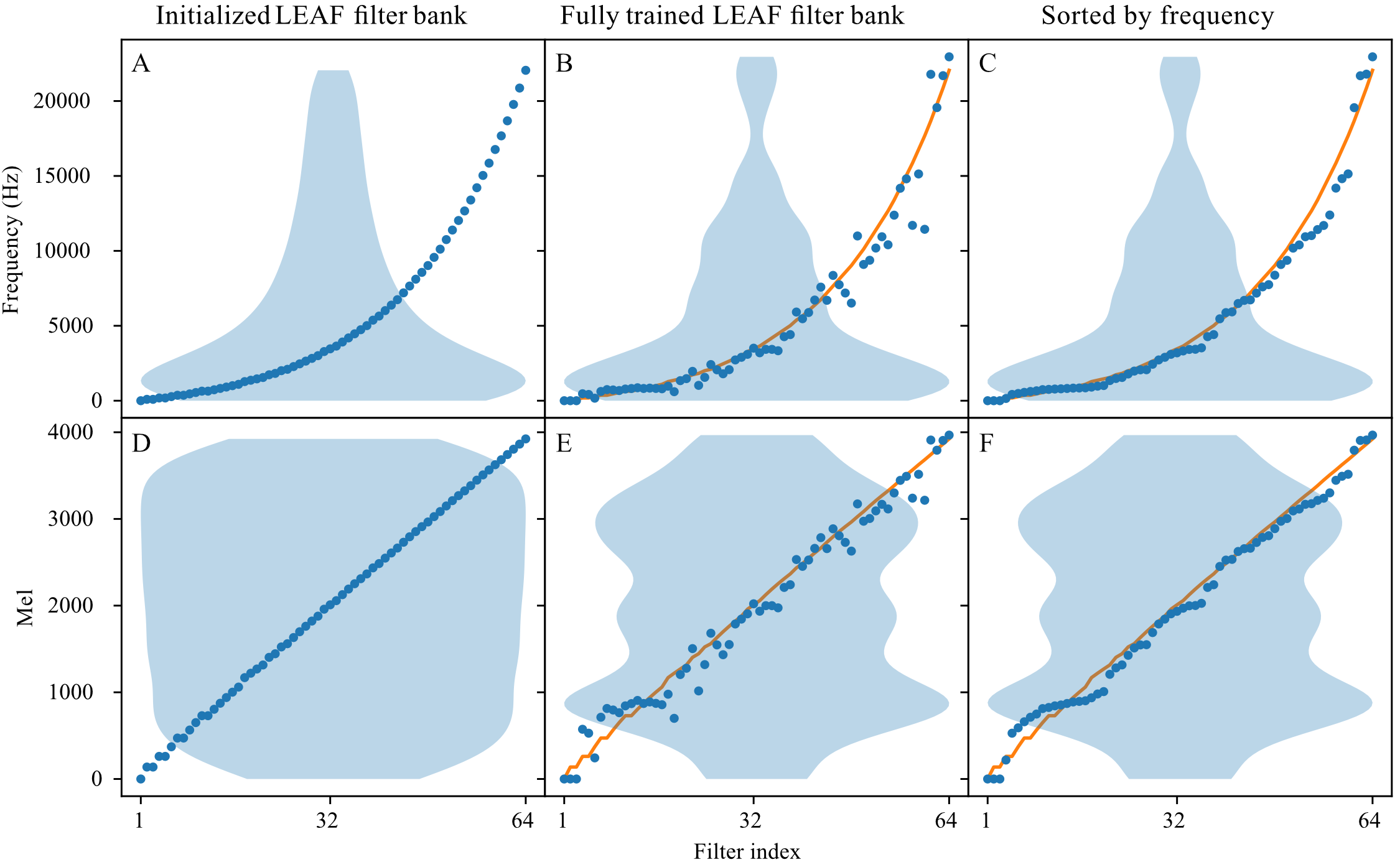

Supplement: S6 Fig — Plots A and D show the initialization curve before training, which is based on the mel scale. Plots B and E show the deviation of each filter from their initialized position after training. Plots C and F show the filters sorted by center frequency, and demonstrate the overall coverage of the frequency range, but do not represent the real ordering in the LEAF representations. Violin plots show the density of filters over the frequency spectrum, the orange line shows the initialization curve for comparison. (TIFF) [file pcbi.1011541.s006.tiff]
