# Supplementary material for: Adaptive representations of sound for automatic insect recognition
Source: PLoS Comput Biol. 2023 Oct 4;19(10):e1011541. doi: 10.1371/journal.pcbi.1011541 (PMC10578591; doi:10.1371/journal.pcbi.1011541)

Comments to the Authors:

Reviewer #1: Very nice paper. Two weaknesses

1) The authors seem to assume that deep learning is the answer. However, it is not clear why that should be true. In the somewhat related problem of classifying mosquitoes by flight sounds, many folk have tried deep learning, but simple nearest neighbor on the right features or a bayes classifier still seems state of the art.

2) The list of many datasets gives the paper a bit of a laundry list feeling.

Thank you. Our set of three datasets represents a progressive expansion using citizen science data; we have tried to clarify and emphasise the structure in the text. See below for our responses regarding features and Bayes classifiers.

“Many insect species produce characteristic sounds that can easily be detected and recorded without large cost or effort.”, Many species, but a very small subset of the number of insect species.

Indeed: acoustic monitoring is not the solution for all insects. In the first two paragraphs of the paper, nevertheless, we motivate developing this tool.

“Using deep learning methods, insect sounds from field recordings could be automatically detected and classified to monitor biodiversity and species distribution ranges.”

True, but you could do it without deep learning too, right? Why the instant jump to deep learning?

Simpler ML methods can work very well in some contexts, such as nearest-neighbour or Bayesian classifiers based on manually-designed acoustic features. However, these methods show their limits in field recordings due to two factors: (a) diverse and loud background and non-target sounds; and especially (b) large numbers of target species to classify [e.g. Salamon et al 2017 10.1109/ICASSP.2017.7952134]. The specific advantage of DL in such cases comes from the automatic optimisation of feature extraction simultaneously with the decision function. We choose to work within the DL paradigm in order to develop methods likely to work in diverse acoustic conditions, with large numbers of target species.

There have been some other research efforts on machine learning of insect sounds using spectrograms [a]. I am not saying you have to compare to these, but it would be at least a good idea to do a more detailed literature search and put your work in context.

Addressed this work on line 91 and added more citations on line 165

“For example, ambient temperature during the recording can influence the frequency..” If you where to classify using a Bayesian classifier, you could condition the classifier on temperature. See the mosquito classification papers of Batista or Keogh etc.

Conditioning a decision on features such as temperature is certainly possible, in Bayesian and non-Bayesian classifiers, both shallow and deep. Our point, in the quoted text, is that such variations complicate the task of feature extraction. We have added a sentence (line 90) to clarify the distinction between these two points.

Note also that the mosquito classification of Batista et al is based on a trap device that records wingbeats *optically*: this successfully yields low-noise data, much lower noise than in general acoustic recordings. This helps to explain why the appropriate algorithm for their data, is not a fully general solution.

In fig 1, it is odd the x-axis limits are 0 to 0.4901. Why not a round number, why that four digit precision?

Changed to 0.5

“compiled from private collections of 186 Orthoptera and Cicadidae recordings (Orthoptera dataset by Baudewijn Odé and Cicadidae 187 dataset by Ed Baker, both unpublished)”

You need to make these available to the reviewers.

All audio material used for training and testing the models is available. These original source datasets from which they were selected are not published, but all recordings that were used for this study are. Are the recordings that were not included in the datasets from these sources due to low quality of interest to the reviewers?

Fig 3 would be so much clearer if the cells with “0.00” were left empty of text.

Fixed this in Figs. 3&4

[a] Yuan Hao, Bilson Campana, Eamonn Keogh. Monitoring and Mining Insect Sounds in Visual Space. SDM 2012

Cited on line 91

Reviewer #2: This was a very well written article. The authors provide a lot of detail making the research accessible to potential readers who might have a strong background within the topic discussed. The flow of ideas was good and the manuscript was easy to read. Here are a few minor suggestions, below. It would be great if the authors could address these minor points which should not take long.

Line 206: could the authors please make it clear if the training, validation and testing files are completely independent, and that two or more spectrograms from a single audio file cannot be found in training/testing. The authors make this statement on the next dataset, but not for InsectSect32.

Clarified on line 250. Within each of our three datasets, audio files are stratified so that training, validation and test sets are independent.

Line 305-311, the authors provide a bit less implementational details related to how they used IRs, compared to the amount of detail provided in general. It would be great if a bit more implementational detail was provided here, so that this matches with the rest of the manuscript.

More detail has been added, on line 364.

Line 360, reference [22] is not the most academic :) Perhaps the authors could find a peer reviewed study that used a simple CNN?

We cite reference 22 (now 28) to give credit for the simple CNN design example that we started from. Other CNN works are cited in our literature review.

Figure 3 (and all the other confusion matrices): please excuse me, but I have some trouble interpreting this plot. Typically, a confusion matrix is normalised (values between 0 and 1), or the raw values are provided. I can't seem to add up the values to one (1) in terms of species X being misclassified as Y, Z , ... along the rows. I'm sure there is a simple explanation, could the authors please clarify.

Added colour bars to Figs. 3&4 to clarify the values

Reviewer #3: The abstract provides a good summary of what was done but would benefit from including some quantitative results. For example, how many species were included in the classifiers? How much better did LEAF do than the standard representation of sound?

Included the maximum number of species in the abstract

Lines 49-51: This sentence makes it sound like stridulation is the only way that sounds are produced by insects. Please modify so that it is clear that this is just one way that insects produce sound.

Modified

Line 61: What are the conventional monitoring methods used for insects? It would be helpful to mention that here.

Added some detail and literature on lines 60-62: [8] Montgomery et al., 2021

Line 66: The phrase ‘since they can function as indicator species’ doesn’t really make sense at the end of this sentence. This seems to be a separate idea related to why it is important to be able to monitor them effectively, not to why they can be monitored acoustically.

Changed the structure of the sentence (lines 64-71). Orthoptera monitoring has been used for evaluating habitat quality and deterioration since they can function as indicator species.

Line 117: There does not need to be a comma after the word ‘parameters’

Changed

Figure 1: Spectrogram B needs labels on the x-axis

Added labels

The introduction provides useful background and explains things well. It would be helpful to also include a brief description of the typical characteristics of sounds produced by Orthoptera and Cicadidae species that are included in the classifiers.

Added a description of the sound characteristics on lines 156-158

Lines 179-180: Please provide more detail about the neural network. Since this and the frontends are central to your results, more information than just a citation needs to be included.

More detail is provided later in the Methods section under “Frontends” and “Network” on lines 393 & 420

Lines 190-191: How did you know what species were in the recordings? Please provide some information about how the recordings were made. What kind of equipment was used to make recordings? Where were the recordings made? Were they collected from wild insects or were they made in a laboratory setting?

Clarified in the text (line 219-220). The recordings were labelled with species names by the recordists. The datasets include field recordings as well as laboratory recordings and were recorded on a wide variety of devices. Species labels from Baker/Ode come with high confidence since they were expert observations, while the species labels from the citizen science sources come with a higher risk of misidentification. Regarding the latter, see also our response below, to the comment regarding lines 213 and 249.

Line 196: Is 40 seconds or 1 minute of data enough to characterize the sounds produced by a species? Please provide some explanation of why you feel confident that this is enough data. I question whether it is a large enough dataset.

Generally it is fairly safe to assume that the repertoire of these insects is much smaller and consistent than the complex vocal repertoire of vocally learning song birds for example, where much more audio material would certainly be needed. Whether the dataset is large enough depends on the application. Since the aim of this work was to compare different audio representation methods, we chose a lower amount of recordings than would be necessary if this was implemented for monitoring applications, to include a more diverse selection of species. For field monitoring purposes, these datasets would have to be expanded to represent all species that occur in the area of interest and more audio material should be gathered.

Lines 213 and 249: I agree that citizen science recordings are a great resource, but when developing classifiers, it is crucial to use recordings from known species. Did you do any quality checking to ensure the accuracy of the species labels for the recordings that you used? If so, please describe this process. If not, please explain why this was not necessary.

We acknowledge the reviewer's concern about the certainty of species identifications in citizen science data. In order to ensure we could be confident about the data we sourced from iNaturalist, we used only research-grade observations, meaning those confirmed to be classified correctly by other validators. The results we present include experiments with and without citizen science data (i.e. InsectSet32 versus InsectSet66), to ensure robustness to this issue. The recordings used in InsectSet32 (that are also partially contained in the two larger datasets) were labelled by experts.

Line 231: recommend changing ‘less species’ to ‘fewer species’

Changed

Lines 274 – 279: This should all be written in the past tense.

Changed

Line 284: Please change ‘can be fed’ to ‘could be fed’

Changed

Line 285: Please define ‘short and rhythmical’ and ‘long and static’. How short is short? How long is long?

Added information and citations on lines 156-158

Line 287-288: would looping short files until they reach 5 seconds not result in over-representing certain sound-types or noise environments?

Looping short files does not result in over-representation, because (a) the unit of analysis and evaluation is each 5-second audio clip as a whole (the process does not inflate the list of data items); and (b) the filters and convolutions are time-shift invariant and do not learn anything 'new' from the within-clip repetition. The method could be applied in principle to variable-length files, and the effect would be formally equivalent. In current deep learning toolkits, this is the standard way to handle short files.

Line 314: why did you not also use torch_audiomentations for the smaller dataset?

For the smaller dataset we implemented offline data augmentation using “audiomentations”, which generates new files from the originals that are then fed into the network. Online data augmentation like “torch_audiomentations” applies the augmentations after the original file has been loaded into the network. The advantage of online augmentation is that it does not use additional storage space and is faster since it is GPU-accelerated. The reason we did not use it for InsectSet32 was that the offline version of “audiomentations” has more capabilities, specifically “FrequencyMask”. “torch_audiomentations” does not include a function that works in an analogous way. With InsectSet47&66 the amount of audio material increased substantially however and offline augmentation was not possible due to storage space limitations. We mention this in the following paragraph starting from line 373, but could expand if necessary.

Lines 370-372: this sentence should be written in the past tense. The paragraph that follows should also be written in the past tense

Changed

Please make re-write the results section so that it is all in the past tense.

Changed

Figure 3 and Figure 4: Please provide a colour scale bar or describe in the figure caption what the different colours in the confusion matrices represent. Also, I assume that the green boxes denote Playtypleura and Myopsalta, but this should be stated in the figure. It would be helpful to have different coloured boxes for each genus for those readers who aren’t familiar with which is which.

Changed

Line 428: Please change ‘less incorrect classifications’ to ‘fewer incorrect classifications’

Changed

Lines 428-431: it would be helpful to state sample sizes for the species that you mention here.

Changed, now line 518

Line 433: I recommend changing ‘run, especially’ to ‘run. In particular,’. Also, when you say ‘significantly better’ – is this statistically significant? If so, please provide the test and p-value. If not, I suggest rewording slightly and using a word other than significantly.

Changed

Line 474: Same comment as above regarding the word ‘significant’

Changed

Line 478: I suggest changing ‘decreased overall’ to ‘was smaller overall’

Changed

Line 479: ‘layers’ should be singular

Changed

Line 503: It’s not clear what you mean by ‘classifications in the opposite direction’

Clarified, this was referring to classifications of Hemiptera as Orthoptera as opposed to Orthoptera as Hemiptera. Now line 610

Line 520: I recommend removing the word ‘managed to’

Removed

Table 5: Is there a digit missing in the lower limit of accuracy for the InsectSet66 dataset?

Changed, I had omitted the last 0 in 0.70

Lines 560-562: could you go into a little more detail about this point? It is an important finding and I feel that it should be discussed in a bit more detail.

Added a short sentence on line 675 to indicate that this will be further discussed in the following paragraphs. Specifically starting from line 708

Lines 579-582: This sentence and much of this paragraph should be written in the past tense.

Changed

Lines 585-586: This seems like a very likely explanation. Have you looked at the sounds produced by some of these species to see if this is true? With the high number of species in the analysis, a detailed look would perhaps be beyond the scope of this paper, but an initial examination and preliminary description of some similarities along with some suggestion for future work based on this would be a nice addition to the manuscript.

Added some more detail and a suggestion for further research starting from line 711

Line 586: I suggest removing the word ‘Apparently’.

Changed

Line 588: I suggest changing ‘less false predictions’ to ‘fewer false predictions’

Changed

Line 591-593: Yes but it is also important to acknowledge here that with a much higher number of species, there is a much lower chance of correct classification by chance and more room for error. So, a similar overall classification success is actually a more successful model when there are more species. You have alluded to this earlier, but I think it is important to bring it up again here.

The lack of difference in performance was in reference to the frontends here (i.e. the performance gap between mel and LEAF), not the datasets. The difference between mel and LEAF was large on InsectSet32, but smaller on InsectSet47&66. Clarified this on lines 706-708

Line 595: I suggest using a word other than ‘significantly’ here.

Changed

Line 597: Should be past tense.

Changed

Line597 and 602: I think the _ should be a 5

Changed

Line 608: Much more closely than what?

Clarified

Line 620: ‘focus’ should be ‘focused’

Changed

Lines 651-652: please provide a bit more detail here on the specific characteristics of insect sounds that may make them less ideal for the use of the mel-scale.

Added on lines 810-812

Lines 668-669: A minimum of 10 recordings per class seems like a reasonable minimum, but what about the duration of those recordings? Is just a few seconds of recordings enough? Please discuss this. Did the amount of data for a species affect classification success? How much data is necessary?

The very small amount of audio material for some species is partly due to the fact that some of these species produce very short sounds. Especially if these sounds occur with longer pauses in between, only short audio clips could be included in these datasets to avoid feeding the networks clips that contain only background noise. A low amount of audio material could be sufficient to represent some of these species. Since this work's focus was to test different audio representations as well as exploring the potential of audio classification using DL on insect sounds, we chose to include these species with low amounts of audio material. But more audio material than this is certainly needed for future implementation in the field and would improve the robustness of the classifiers.

Below are some plots showing the effects of number of recordings and length of audio material on the classification accuracy. The classification accuracy is derived from the best performing LEAF run on InsectSet66 (Table 4, Suppl. Fig. 4).

Fig. 1: Number of recordings per species vs. classification accuracy:

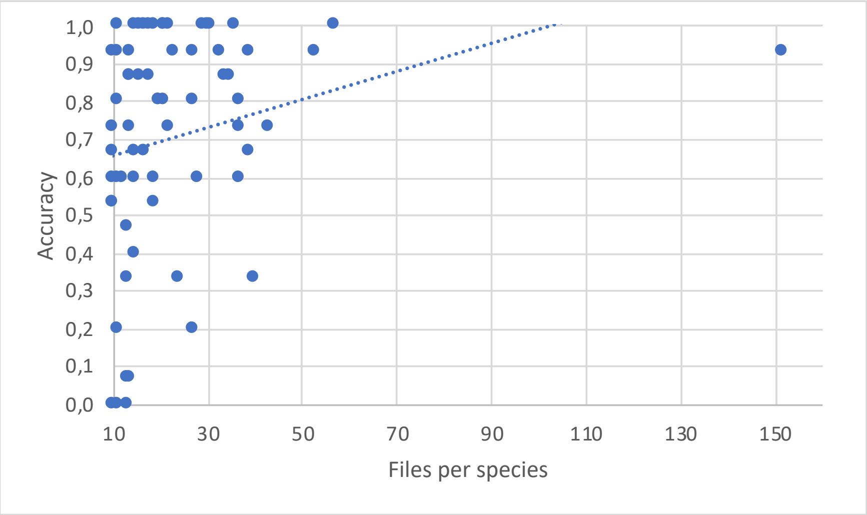

Fig. 2: Seconds of audio material per species vs. classification accuracy:

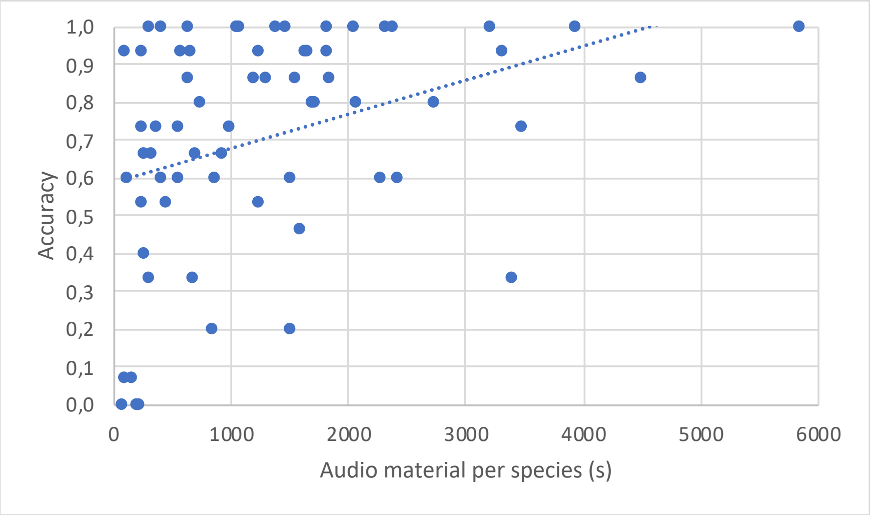

Supplement: Attachment — Submitted filename: Rebuttal_letter.pdf [file pcbi.1011541.s007.pdf]
